# Fabrication and properties of PLA/β-TCP scaffolds using liquid crystal display (LCD) photocuring 3D printing for bone tissue engineering

**DOI:** 10.3389/fbioe.2024.1273541

**Published:** 2024-02-19

**Authors:** Boqun Wang, Xiangling Ye, Guocai Chen, Yongqiang Zhang, Zhikui Zeng, Cansen Liu, Zhichao Tan, Xiaohua Jie

**Affiliations:** ^1^ School of Materials and Energy, Guangdong University of Technology, Guangzhou, Guangdong, China; ^2^ School of Intelligent Manufacturing, Dongguan Polytechnic, Dongguan, Guangdong, China; ^3^ Dongguan Hospital, Guangzhou University of Chinese Medicine, Dongguan, Guangdong, China; ^4^ The Second Clinical College of Guangzhou University of Chinese Medicine, Guangzhou, Guangdong, China; ^5^ Foshan Hospital of Traditional Chinese Medicine, Guangzhou University of Chinese Medicine, Foshan, Guangdong, China; ^6^ Affiliated Hospital of Jiangxi University of Chinese Medicine, Nanchang, Jiangxi, China

**Keywords:** polylactic acid, beta-tricalcium phosphate, liquid crystal display, 3D printed scaffolds, bone tissue engineering

## Abstract

**Introduction:** Bone defects remain a thorny challenge that clinicians have to face. At present, scaffolds prepared by 3D printing are increasingly used in the field of bone tissue repair. Polylactic acid (PLA) has good thermoplasticity, processability, biocompatibility, and biodegradability, but the PLA is brittle and has poor osteogenic performance. Beta-tricalcium phosphate (β-TCP) has good mechanical properties and osteogenic induction properties, which can make up for the drawbacks of PLA.

**Methods:** In this study, photocurable biodegradable polylactic acid (bio-PLA) was utilized as the raw material to prepare PLA/β-TCP slurries with varying β-TCP contents (β-TCP dosage at 0%, 10%, 20%, 30%, 35% of the PLA dosage, respectively). The PLA/β-TCP scaffolds were fabricated using liquid crystal display (LCD) light-curing 3D printing technology. The characterization of the scaffolds was assessed, and the biological activity of the scaffold with the optimal compressive strength was evaluated. The biocompatibility of the scaffold was assessed through CCK-8 assays, hemocompatibility assay and live-dead staining experiments. The osteogenic differentiation capacity of the scaffold on MC3T3-E1 cells was evaluated through alizarin red staining, alkaline phosphatase (ALP) detection, immunofluorescence experiments, and RT-qPCR assays.

**Results:** The prepared scaffold possesses a three-dimensional network structure, and with an increase in the quantity of β-TCP, more β-TCP particles adhere to the scaffold surface. The compressive strength of PLA/β-TCP scaffolds exhibits a trend of initial increase followed by decrease with an increasing amount of β-TCP, reaching a maximum value of 52.1 MPa at a 10% β-TCP content. Degradation rate curve results indicate that with the passage of time, the degradation rate of the scaffold gradually increases, and the pH of the scaffold during degradation shows an alkaline tendency. Additionally, Live/dead staining and blood compatibility experiments suggest that the prepared PLA/β-TCP scaffold demonstrates excellent biocompatibility. CCK-8 experiments indicate that the PLA/β-TCP group promotes cell proliferation, and the prepared PLA/β-TCP scaffold exhibits a significant ability to enhance the osteogenic differentiation of MC3T3-E1 cells *in vitro*.

**Discussion:** 3D printed LCD photocuring PLA/β-TCP scaffolds could improve surface bioactivity and lead to better osteogenesis, which may provide a unique strategy for developing bioactive implants in orthopedic applications.

## 1 Introduction

Bone defects are a severe health problem worldwide and remain a significant challenge facing orthopedists (Liu et al., 2023). Clinically, the main methods to repair bone defects include bone transplant surgery, Masquelet technology, Ilizarov bone transfer technology, tissue engineering bone technology, gene therapy, and other treatment methods (Nauth et al., 2018; Mathieu et al., 2022). At present, bone grafts or substitute materials are usually implanted in bone defects for treatment (Su et al., 2023). Unfortunately, current clinically-used bone grafts or bone graft substitutes are insufficient to meet the needs of repairing complex bone defects (de Silva et al., 2023). Therefore, developing bone graft materials with high osteogenic efficiency is not only of great clinical significance but also a scientific problem that needs to be solved urgently.

Tissue engineering refers to the use of basic principles and technologies of cell biology and materials science to construct and cultivate substitute materials with physiological functions *in vitro* to repair defective tissues so that they can compensate for or replace the functional activities related to the repaired tissues and organs. Bone tissue engineering (BTE) refers to the use of principles and technologies of cell biology and materials science to promote cell migration, growth and proliferation through the introduction of exogenous scaffolds and the continuous supply of regulatory growth factors, ultimately achieving the repair of bone defects (Xie et al., 2021). In recent years, various advanced bone tissue engineering scaffolds have been clinically designed to treat bone defects (Bunpetch et al., 2019; Fernandes et al., 2019; Zhou et al., 2019; Wang et al., 2022; Wang et al., 2022; Ye et al., 2022). The preparation of tissue engineering scaffolds by 3D printing technology can realize the control of material pore size, porosity, and permeability, accurately simulate the shape of human bones and apply it to bone tissue repair and reconstruction, providing a promising treatment for repairing bone defects method (Mirkhalaf et al., 2023). At present, the most widely used 3D printing methods for bone tissue engineering mainly include fused deposition modeling (FDM), selective laser sintering (SLS), and stereolithography (SLA).

SLA is a technology that builds objects by printing liquid photosensitive resin layer by layer based on a digital model. The advantages of SLA include a short production cycle, a vast production field and high printing precision (Li et al., 2023). However, large volumes, low space utilization, and expensive 3D printing equipment limit the application of SLA in bone tissue engineering (Mirkhalaf et al., 2023). Liquid crystal display (LCD) technology is an emerging 3D printing technology for UV curing moulding of photosensitive resin (Zhu et al., 2022). Compared with the early SLA printing technology, LCD technology can achieve fast molding speed and high printing accuracy simultaneously and is more controllable on the size and surface structure of the scaffolds (Shan et al., 2020; Madarevi and Ibri, 2021; Sotov et al., 2021).

In recent years, a variety of materials including UHMWPE, PEEK, Chitosan, GelMA, PCL, Zein, and many other biomaterials for bone implants (Herrera-Ruiz et al., 2022; Mamidi et al., 2022; Mamidi et al., 2023). PLA can be produced from nontoxic renewable feedstock and PLA is biodegradable, gradually breaking down into non-toxic lactic acid in the body and metabolized by the human body (Singhvi et al., 2019). This makes PLA suitable for applications where gradual replacement by new tissue is desired without the need for a second surgery to remove the implant. PLA exhibits good compatibility with human tissues, reducing the likelihood of immune reactions or rejection (Ranakoti et al., 2023). This contributes to minimizing postoperative complications and promoting the healing of the implant. The good biocompatibility and biodegradability of PLA have made it approved by the U.S. Food and Drug Administration (FDA) as a biomedical material (Li et al., 2020). PLA has found widespread use in the medical field, especially in clinical validations related to bone implantation. Therefore, polylactic acid (PLA) is considered one of the most promising biopolymers. However, defects such as low mechanical strength, poor toughness, and insufficient biological activity of PLA limit its further application in bone tissue engineering (Ye et al., 2022).

Tricalcium phosphate (β-TCP) is a synthetic ceramic material with excellent biodegradability and biocompatibility, which has been widely used in bone tissue engineering (Boda et al., 2023). β-TCP not only has good mechanical properties and can be used as a reinforcing material to significantly improve the mechanical strength of composite materials, but more importantly, β-TCP also has excellent osteoconductivity and inductivity, which can promote cell proliferation and differentiation (Lu et al., 2023; Silva-Barroso et al., 2023). However, preparing scaffolds with controllable biomimetic orientation microporous structures on the surface has always been a complex problem in preparing scaffolds from PLA-based composite materials. In the currently widely used piston extrusion method, due to the swelling phenomenon of polymer materials, the texture of the extruded fiber disappears after being formed through the processed micro-groove nozzle, and the LCD light-curing technology has micron-level layer thickness parameters. The preparation of the scaffold fiber in a non-extrusion way, combined with the slurry prepared by photocuring bio-based, can realize liquid molding. Therefore, this work aimed to prepare PLA/β-TCP scaffold that scaffolds that have not only high precision, texture, and roughness on the fiber surface but also have outstanding biological properties for bone tissue engineering.

**SCHEME 1:**
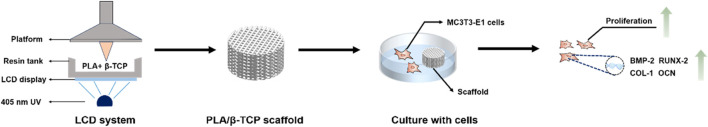
Schematic illustration of the PLA/β-TCP scaffolds using LCD photocuring 3D printing in bone tissue engineering. With the help of a computer and a display screen, ultraviolet light passes through the transparent area to illuminate the photosensitive resin in the resin tank. The PLA/β-TCP resin material is exposed and solidified layer by layer into a 3D scaffold.

To this end, unlike most other 3D printing methods, we prepared 3D-printed PLA/β-TCP scaffolds via LCD photocuring 3D printing. The prepared PLA/β-TCP scaffold has a porous structure, and its mechanical properties are also satisfactory. *In vitro* experiments further investigated the biological properties of the PLA/β-TCP scaffold, including its biocompatibility and osteogenic differentiation properties. We found that the prepared PLA/β-TCP scaffold not only had no toxicity to MC3T3-E1 cells but also promoted their proliferation, and related experiments further confirmed that it had excellent osteogenic properties (Scheme 1). Consequently, the designed LCD photocuring PLA/β-TCP scaffolds could represent a promising substitute material in bone tissue engineering to repair bone defects.

## 2 Materials and methods

### 2.1 Material

LCD Photocurable Bio-based Polylactic acid (PLA) was purchased from Yisheng New Material Co., Ltd. (Xiaogan, China). Tricalcium phosphate powder (β-TCP, particle size: 1.2 μm) was obtained from Epurui Material Co., Ltd. (Nanjing, China). Dispersant: Silane coupling agent (KH-550) was purchased from Trands Chemical Additives Co., Ltd. (Nanjing, China). Anhydrous ethanol was purchased from Sinopharm Chemical Reagent Co., Ltd. (Beijing, China).

### 2.2 Preparation of the scaffolds

#### 2.2.1 β-TCP modification

Firstly, 100 mL of absolute ethanol, 5 mL of silane coupling agent, and 50 g of β-TCP powder were added into a beaker, then stirred in a water bath at 50°C for 6 h, centrifugally filter, dry in a vacuum at 55°C for 24 h, and then grind to obtain β-TCP ceramic powder.

#### 2.2.2 Preparation of PLA/β-TCP slurry

The β-TCP powder was added to the light-cured bio-based PLA solution in a beaker, vibrated and stirred for 30 min under an ultrasonic washing machine, and then mixed with a planetary ball mill at a speed of 300 rpm for 6 h, putting the ceramic slurry obtained after grinding and blending into a vacuum defoamer for degassing, and finally filtered it with a sieve The beads were ball milled to get a 3D printed PLA/β-TCP ceramic slurry with uniform composition, which was stored in the dark for future use.

#### 2.2.3 Fabrication of the PLA/β-TCP scaffolds

Three-dimensional cylindrical scaffolds were designed using SolidWorks software (Dassault Systemes S.A, France). The structure and size of the printed bio-scaffold were shown in Supplementary Figure S1. Use CHITUBOX (CBD-Tech, China) software to slice the scaffold model. The specific parameters were shown in Supplementary Table S1. After completion, import the setting parameters to the computer. Pour the PLA/β-TCP printing material into the resin tank of the high-precision LCD-3D printer (Chuangxiang LD-002R, China) and click to start printing. A 3D scaffold with a diameter of 10 mm, a height of 6 mm, a fiber diameter of 400 μm, and a left and right gap of 400 μm was successfully prepared. Then put the scaffold in an ultrasonic cleaner for 20 min, dry it with a hair dryer, and put it into a secondary curing machine to cure for 15 min before use.

### 2.3 Characterization of the scaffolds

#### 2.3.1 Rheological properties test of PLA/β-TCP slurry

Obtain representative samples of the material and mix of the material to eliminate any unevenness. The rheological properties of different PLA/β-TCP slurries were tested at room temperature using a Modular Compact Rheometer (MCR702, Anton-Paar, Austria). Set the applicable temperature to −30∼300°C, the rotation speed 0∼3,000 r/min, and the shear rate 0.01∼4,000 s-1'. Operate the rheological instrument to conduct the test and record data.

#### 2.3.2 Scanning electron microscopy (SEM) analysis

Prepare the sample for observation and apply a gold coating. Place the sample on the sample stage of SEM, then use SEM (EM-30, COXEM, South Korea) to observe the structure, surface, and compression fracture morphology of the 3D-printed scaffold.

#### 2.3.3 Confocal laser microscope (CLSM) analysis

Fix the sample on the sample stage of the CLSM (KEYENCE, VK-X1100, Tokyo, Japan), use the CLSM to adjust the focus and start the confocal laser microscope to scan and obtain an image of the sample surface, and finally reconstruct the three-dimensional image of the obtained image.

#### 2.3.4 Porosity analysis

The porosity of the scaffolds was determined by the immersion medium method. First, calculate the volume of the scaffold, weigh the mass (m_1_) of the sample in the air with an electronic balance, then immerse it in absolute ethanol to make it saturated, soak it for a certain period, and then take out the sample, carefully wipe off the medium on the surface with filter paper, and then weigh its total mass (m_2_) in the air, and the porosity is calculated according to formula (1).

$$P=\left[\left(m2-m1\right)/\rho V\right]\times 100\%$$ (1)

*P* is the porosity, %; *ρ* is the density of the liquid medium, g/cm^3^; *V* is the volume of the sample, cm^3^.

#### 2.3.5 Mechanical properties tests

According to GB/T 1041-2008, prepare the sample to be tested, install the sample on the fixture of the electronic universal testing machine (AGS-X, Shimadzu Corporation) using the appropriate fixture, set the testing parameters of the testing machine, and start the testing machine to perform the compressive strength of the scaffold sample.

#### 2.3.6 XRD analysis

β-TCP powder and PLA/10% β-TCP scaffold were measured using an X-ray diffractometer (MiniFlex600, Rigaku, Japan). Prepare the sample for testing, load it onto the sample stage of the XRD instrument, select the X-ray radiation source (Cu Kα, λ = 1.5406 Å), set the scanning range of 20°–70° and scanning rate of 4°/min on the XRD instrument, initiate the scan, and record the data.

#### 2.3.7 Degradation of scaffolds

The degradation and pH values of the scaffolds were studied in the SBF for about 6 weeks. The scaffolds were weighed (m_1_) and then immersed in a centrifuge tube containing 10 mL of SBF at 37°C. After rinsing the scaffolds with distilled water, the scaffolds were dried until weight stabilization and then weighed (m_2_) for a total of 6 weeks. Calculate the degradation rate according to the following formula: (m_1_–m_2_)/m_1_×100%. The pH value of the degradation medium was measured by a pH meter (Mettler Toledo).

### 2.4 Biocompatibility of scaffolds

#### 2.4.1 Cell culture

Mouse embryonic osteoblast MC3T3-E1 cells (The cell lines present in this study were obtained from Cyagen Biotechnology) were thawed and centrifuged, and then the MC3T3-E1 cell suspension in the centrifuge tube was inoculated into a cell culture flask, and MEM-α medium (containing 10% FBS, 1% penicillin and strep to Mycin) (Gibco, USA), and then cultured in a cell incubator (5% CO_2_, 37°C).

#### 2.4.2 Cell proliferation

MC3T3-E1 cells were seeded into 24-well plates with scaffolds at a concentration of 5 × 10^3^/mL, and the 24-well plates were incubated at 37°C for 24 h. After co-culturing for 1, 3, and 5 days, Cell Counting Kit-8 solution (CCK-8, Biosharp, China) was added to each 24-well and incubated for 2 h. After incubation, use a full-wavelength microplate (Multiskan GO, Thermo Fisher Scientific, USA) to measure the OD value of all wells at a wavelength of 450 nm.

#### 2.4.3 Live/dead cell staining

A live/dead staining assay was used to determine the cytotoxicity of scaffolds. MC3T3-E1 (5×10^3^) cells were seeded into 24-well plates with scaffolds at 37°C for 24 h. After co-culturing for 1, 3, and 5 days, discard the culture medium and wash twice with phosphate-buffered saline (PBS, Gibco, USA), then stain with Calcein-AM/PI kit (Bestbio, China) for 20 min. After washing with phosphate-buffered saline (PBS, Gibco, USA) twice, the cells were imaged using a fluorescence microscope (Leica, Germany).

#### 2.4.4 Hemolysis tests

The collected healthy rat blood (4 mL) containing ethylenediaminetetraacetic acid (EDTA) gifted from another research group was diluted with PBS (5 mL). Scaffolds were then incubated in PBS for 30 min at 37°C, followed by 0.2 mL of diluted blood co-cultured with scaffolds. Positive and negative control groups were set with deionized (DI, Gibco, USA) water and PBS, respectively. The scaffolds of all groups were incubated at 37°C for 30 min, and the OD value of the supernatant was measured at 545 nm after centrifugation. The hemolysis ratio (HR) was calculated, as we previously reported (Ye et al., 2018).

### 2.5 Osteogenic activity of the scaffolds *in vitro*

#### 2.5.1 Alkaline phosphatase (ALP) staining assay

The osteogenic differentiation potential of MC3T3-E1 cells on scaffolds was determined by ALP staining assay. Inoculate the MC3T3-E1 cell suspension with a concentration of 1 × 10^4^/mL in a 6-well plate, and then the scaffolds were incubated with osteogenic medium culture (Cyagen Biosciences, USA) for 7 and 14 days. After the induction culture, discard the medium, add PBS to wash 3 times, fix the cells with 4% paraformaldehyde at room temperature for 30 min, then wash 3 times with PBS, add ALP stain working solution (Beyotime Biotechnology, China) for 6 h, then wash 3 times with PBS, observed and collected images under a microscope (Leica, Germany).

#### 2.5.2 Alkaline phosphatase (ALP) activity

MC3T3-E1 cells and the scaffolds were incubated with osteogenic medium culture for 7 and 14 days according to method 2.5.1. Then, 0.2% Triton X-100 (Sigma-Aldrich, USA) was added to each well, pipetting repeatedly for 30 min, and placed in a 4°C refrigerator for 24 h. ALP detection kit (Beyond Biotechnology, China) was used to detect ALP activity and the BCA protein assay kit (Pierce, USA) was used to detect total protein. Relative ALP activity was calculated based on the previous calculation method (Li et al., 2020).

#### 2.5.3 Alizarin red stained and activity assay

MC3T3-E1 cells and the scaffolds were incubated with osteogenic medium culture for 7 and 14 days according to method 2.5.1. After the induction culture, the cells were fixed with 4% paraformaldehyde, added Alizarin red S staining solution (Solarbio, China) for 60 min, removed staining working solution, then washed with PBS, observed and collected images under a microscope. Added Cetylpyridinium Chloride (CPC) to Disodium Hydrogen Phosphate (Sodium dihydrogen Phosphate, Na_2_HPO_4_) to prepare a 10% CPC solution. After drying, the prepared CPC solution to each well, and its OD value at 562 nm.

#### 2.5.4 Expression of osteogenic differentiation-related genes

To detect the expression of osteogenesis-related genes in MC3T3-E1 cells on the scaffolds, MC3T3-E1 cells, and the scaffolds were incubated with osteogenic medium culture for 7 and 14 days according to the method of 2.5.1. The total RNA of each group was extracted and reverse-transcribed into cDNA. Finally, the expression levels of osteogenesis-related genes in each group were evaluated by real-time semiquantitative polymerase chain reaction (RT-qPCR). Briefly, the experimental operation steps refer to our previous research (Li et al., 2020). The osteogenic-related gene expressions, including BMP-2, OCN, Runx-2, and COL-1 were measured. Glyceraldehyde-3-phosphate dehydrogenase (GAPDH) was used as the housekeeping gene and the relative gene expression was calculated using the 2^−ΔΔCt^ method. The sequences of primers were shown in Supplementary Table S2.

#### 2.5.5 Immunofluorescence staining

To evaluate the osteogenesis effect of scaffolds *in vitro*, the expression levels of Runx-2 factors were invested by immunofluorescence staining. Briefly, the MC3T3-E1 cell and the scaffolds were incubated with osteogenic medium culture for 7 days. The cells were fixed with 4% paraformaldehyde for 30 min and washed with PBS. Added 0.5% Triton X-100 to cells at room temperature for 5 min and then washed PBS. Afterward, the cells were blocked with 5% bovine serum albumin (BSA, BioFroxx) for 30 min at room temperature. Discarded the blocking solution, washed with PBS, added the primary antibody of Runx-2 (1:100 dilution; Abcam, USA) to each well plate and incubated overnight at 4°C. After washing, the secondary antibody (1:200 dilution; EarThox, USA) was added and incubated at room temperature in the dark for 2 h. Finally, DAPI staining solution was added to counterstain the cell nuclei for 5 min. After washing with PBS, the immunofluorescence staining images were observed and collected. Fluorescence intensity was evaluated using ImageJ software (NIH, USA).

#### 2.5.6 Elisa assay

The BMP-2 of the osteoblastic cells was assayed with an ELISA kit (Elabscience Biotechnology Co., Ltd., China) according to the manufacturer’s instructions. Briefly, the MC3T3-E1 cell and the scaffolds were incubated with osteogenic medium culture for 7 days and 14 days according to method 2.5.1. According to the kit instructions, the culture plates were pre-coated with antibodies specific to the protein marker. Add samples and standards and react at 37°C for 90 min. Add biotinylated detection antibody specific for BMP-2 protein markers and react at 37°C for 60 min. The processing work was carried out at 37°C for 30 min. Wash 5 times with 1X wash buffer. TMB reacts at 37°C for 20–25 min. Add stop solution and read OD at 450 nm.

### 2.6 Statistical analysis

All data were analyzed by SPSS 25.0 software. All data are expressed as mean ± standard deviation. Statistical comparisons among groups were evaluated by one-way ANOVA analysis. *p* < 0.05 was considered statistically significant, and *p* < 0.01 was considered highly statistically significant.

## 3 Results and discussion

### 3.1 Rheological characterization

**FIGURE 1:**
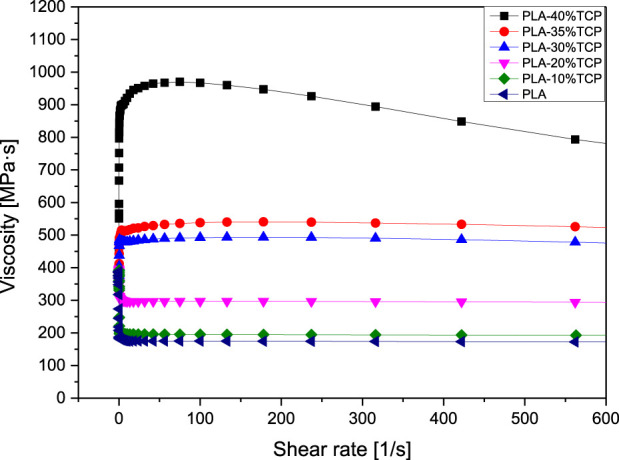
Rheological curve of PLA/β-TCP slurry.

Rheology is the study of the flow of matter, and the flow speed of 3D printed materials is of great significance to the final object, especially in the bioprinting process. Figure 1 showed the Rheological Curve corresponding to the slurry. It can be seen that the viscosity of PLA, PLA/10%β-TCP, and PLA/20%β-TCP slurry decreased with the increase of shear rate, showing the phenomenon of shear thinning, indicating that slurry with different solid content had non-Newtonian fluid properties, and theoretically meets the printing requirements. But with the increase of the β-TCP component, the viscosity of PLA/30%β-TCP and PLA/35%β-TCP slurry first increased with the addition of shear rate and then slowly decreased. This was mainly because the number of β-TCP particles dispersed in the slurry increased, the relative content of organic media in the slurry decreased, and the probability of friction and collision between particles increased during slurry under shear stress. When the concentration keeps increasing and approaches the tightest alignment, the relative motion between the two layers will make the particles deviate from the closest alignment and increase their volume, which requires extra energy consumption. In the initial stage, it was shown as the increase of slurry viscosity. When the shear rate increased to 80 s^-1^, the PLA matrix was still the primary fluid after the ceramic particles were spread out. The density exhibits pseudoplastic fluid properties and slowly decreases.

### 3.2 Preparation and characterization of the scaffolds

**FIGURE 2:**
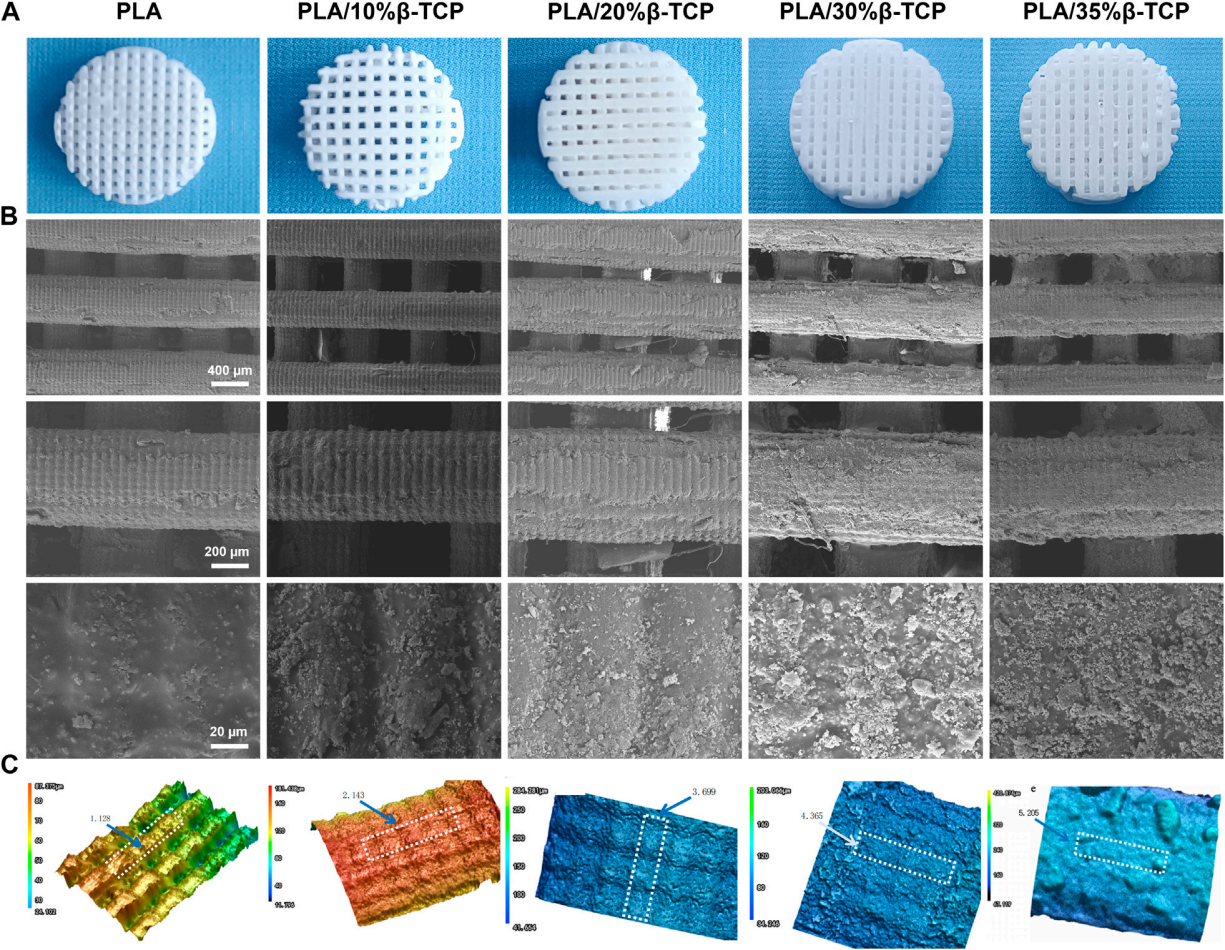
Morphology of the 3D-printed scaffolds. **(A)** 3D printed PLA/β-TCP scaffolds with different proportions. **(B)** SEM images of the 3D-printed scaffolds, respectively. Scale bar = 400 μm, 200 μm and 20 μm. **(C)** The three-dimensional morphology of the scaffold fiber surface and the surface roughness of the selected area Ra (mean arithmetic height) (μm).

We successfully fabricated customized-designed scaffolds by LCD photocuring 3D printing. First, stir the slurry with different proportions for 10 min, pour it into the trough of the 3D printer, set the parameters according to Supplementary Table S1, and start the LCD light-curing 3D printer. The β-TCP slurry will be light-cured under the exposure of the smooth surface (band = 405 nm). Reaction and single-layer curing molding on the substrate. After replenishing the slurry, the substrate was pressed down on the surface exposure film again, and the next layer of the layered model image was continued. After layer-by-layer curing and superimposition, a scaffold with a through structure was prepared. After taking out the scaffold Rinse the surface of the scaffold with absolute ethanol, and finally perform secondary curing in a UV light box for 15 min. The prepared scaffold was shown in Figure 2A. PLA/40%TCP failed to form after many times of printing. This is because during the forming process, the high-viscosity slurry will naturally agglomerate under the action of surface tension, which is not conducive to the spreading of the uniform material layer, and it is easy to cause edge defects. Therefore, the higher the viscosity of the slurry, the easier it is to form the above shortcomings, and it will affect the molding accuracy.

The surface morphology and roughness of 3D-printed scaffolds are essential factors affecting cell adhesion and directional differentiation. SEM was employed to observe the morphologies of different scaffolds. Figure 2B shows the macrostructure and the microscopic surface morphology of different scaffolds, and Figure 2C shows the three-dimensional morphology and roughness of the scaffolds under a laser confocal microscope. As shown in Figure 2A, all scaffolds had a three-dimensional network structure, which could facilitate the exchange of oxygen and nutrients. The surface of the scaffolds cured by LCD light 3D printing showed “bark-like” lines and the pure PLA microgrooves were prominent. The density increases with the increase of β-TCP content, and the structure is in grooves or micro-holes. Selected area electron diffraction analysis was carried out on the particles on the surface of the scaffold. The TEM photos and diffraction patterns were shown in Supplementary Figure S2. The results conformed to the characteristics of the hexagonal crystal structure. Combined with the lattice constant analysis and analysis, it was confirmed that the particles were β-TCP. The texture structure of the surface of the scaffold was determined by the micron controllable layer thickness parameter during exposure, and the regular rough surface was determined by the particle size of β-TCP. The particles uniformly mixed in the slurry are formed together when the PLA cures. This controllable structure has good application value for cell adhesion and directional differentiation, which cannot be realized by current bio-extrusion 3D printing technology.

Supplementary Figure S3 shows the porosity of the scaffold calculated by the medium soaking method. The porosity of the PLA scaffold is 48.6%, close to the theoretical value of 50%. The reason for the deviation is that the upper and lower fibers are better bonded during the printing process. The actual spacing set is 0.38 mm, which is smaller than the theoretical spacing of 0.40 mm, which is equivalent to the increase in the natural packing volume in formula (1), resulting in a decrease in the porosity of the scaffold. As the amount of β -TCP increased, more β-TCP particles adhered to the surface of the scaffold, which partially occupied the gaps of the porous scaffold, resulting in a decrease in porosity.

EDS analysis was performed on the PLA/β-TCP scaffolds containing different proportions of β-TCP, as shown in Supplementary Figure S4. The results showed that: with the increase of the mass fraction of β-TCP in the scaffold, the mass of Ca^2+^ particles attached to the surface of the scaffold fibers accounted for ratio increases, but their ratios are all smaller than the β-TCP content of the corresponding scaffold, forming a complementary relationship between the mass ratio and the surface morphology.

**FIGURE 3:**
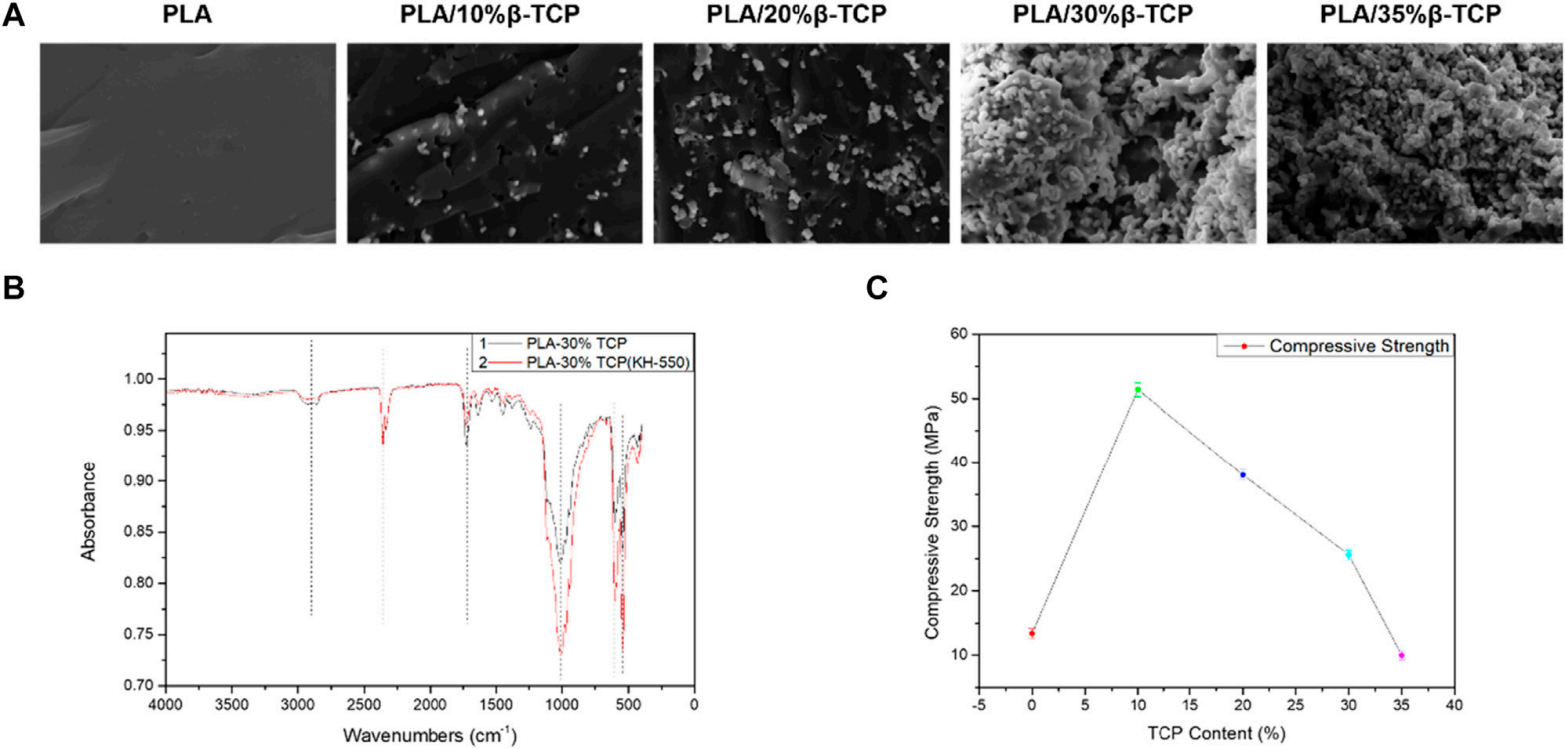
Mechanical performance of the 3D-printed scaffolds. **(A)** SEM morphology of the fractured section of the different 3D-printed scaffold under compression. **(B)** FTIR spectra of 3D-printed PLA/30%β-TCP scaffold and PLA/30%β-TCP scaffold (KH-550). **(C)** The compression strength of the different 3D-printed scaffolds.

Figure 3A shows the cross-sectional morphology of the biological scaffold after the compression test. It can be seen that as the proportion of β-TCP increases to 30%, its particles can be well distributed in the PLA matrix, and the bonding interface between the two was better. It can be seen from the infrared spectrum in Figure 3B that the infrared characteristic absorption peak of the leading group of PLA-30%β-TCP added with the silane coupling agent KH-550 was significantly enhanced due to the silane group of the silane coupling agent or the hydrolyzed silicon hydroxyl group integrates with the hydroxyl group on the surface of the inorganic substance. In contrast, the other carbonyl organic group forms a covalent bond with the organic polymer compound, which can couple the interface of two materials with very different properties, thereby improving the dispersion and properties of the particles’ slurry fluidity and enhancing interfacial adhesion. When the ratio reaches 35%, the distribution of β-TCP particles per unit area on the cross-section increases continuously, and the probability of particle agglomeration also increases. Cavitation, fracture separation along matrix and grain boundaries. Excellent mechanical properties are essential for scaffolds. To investigate the mechanical properties of different scaffolds, we performed a compression test on PLA and PLA/β-TCP scaffolds. Figure 3C showed the compressive strength of the PLA/β-TCP scaffold. It can be seen that the compression strength of the scaffold can be improved with the appropriate addition of β-TCP. With the increase in the dosage of β-TCP, the compression strength of the scaffold increases first and then decreases. When the ratio of β-TCP is 10%, the maximum value is 52.1 MPa, and the strain is 31.4%. This is because the elliptical structure of β-TCP ceramic particles could withstand the load in the system and had high compressive strength. When the appropriate amount was added to the PLA matrix for uniform dispersion, β-TCP can absorb and transfer the external compression force during the compression process, thus improving the load capacity per unit of stress area. However, when the amount of β-TCP increased, particle aggregates were easy to form in the slurry system, and the binding force between them was minimal. After solidification, micro-crack sources will be included in the material. When the biological scaffold was squeezed, these micro-cracks will expand and reduce the compressive strength of the system. The incorporation of polymers into a mixture to enhance its performance is a feasible approach. Mohan A et al. synthesized PHB-based nanocomposites using nanomixtures and nanoclay along with modified montmorillonite (MMT) as filler. The results show that the PHB/OMMT composite blends exhibited augmented mechanical properties compared to neat PHB (Mohan et al., 2021). Therefore, in the process of 3D printing PLA/β-TCP paste based on LCD technology, adding the appropriate amount of β-TCP can greatly improve the compression performance of the scaffold. However, excessive dosage could easily cause agglomeration and reduce the compressive strength of the material. Therefore, we studied the *in vitro* biocompatibility and osteogenic properties of PLA/10%β-TCP scaffolds.

X-ray diffraction analysis was performed on β-TCP powder and PLA/10%β-TCP scaffold. As shown in Supplementary Figure S5, by comparing with the standard β-TCP diffraction pattern, β-TCP appeared in the XRD pattern of PLA/10%TCP scaffold. The characteristic diffraction peaks (main strong peak 2θ = 31.02°, secondary strong peak 2θ = 34.33°), comparing the X-ray diffraction patterns of β-TCP powder and PLA/10%β-TCP scaffold, most of the peak positions have not changed, which shows that during the preparation process of the 3D printed scaffold, no phase change of β-TCP occurred, and no new diffraction peaks appeared, indicating that no new impurities were generated during the preparation process.

**FIGURE 4:**
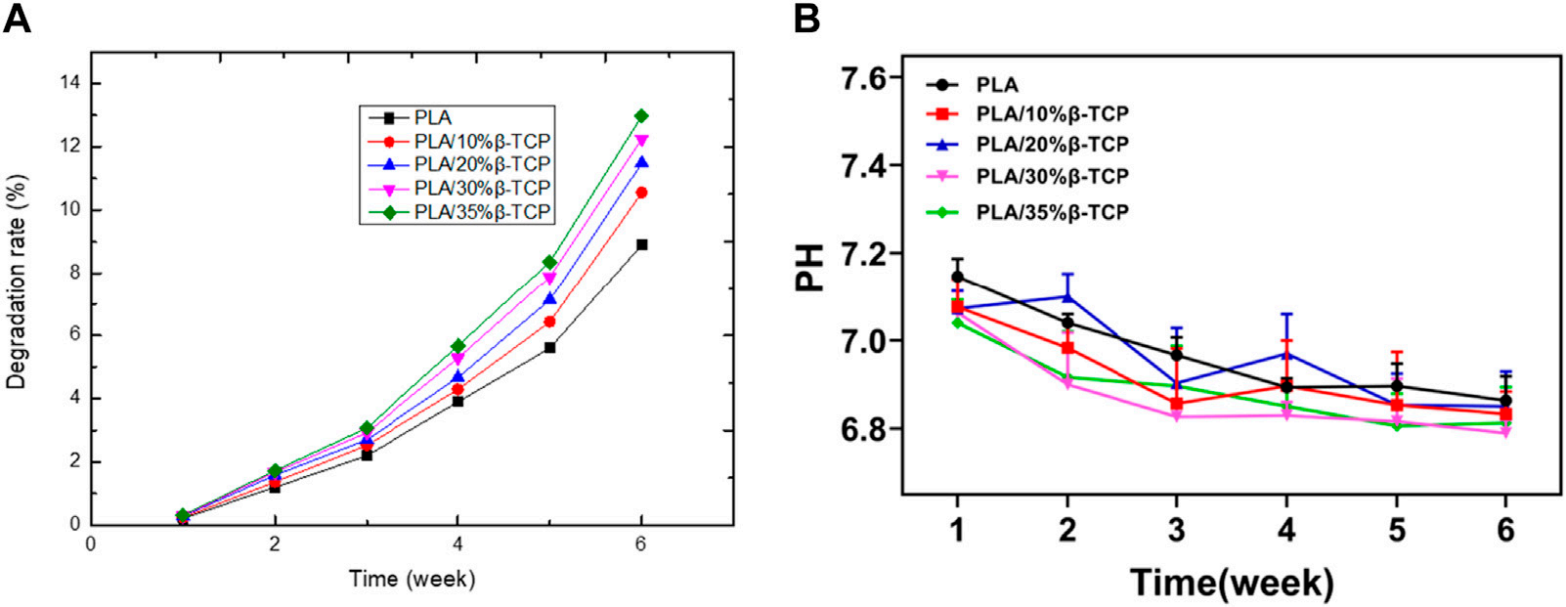
The degradation of the scaffold. **(A)**Weight of scaffolds after degradation *in vitro*. **(B)** pH value in the SBF.

Figure 4A showed the degradation rate curve of the scaffolds in SBF at different times. It can be seen that as the degradation time prolongs, the degradation rate of the scaffolds gradually increases, but the period of rapid mass loss mainly occurs after the 3rd week. Among them, the PLA/35%β-TCP scaffold degraded the most in the 6th week, with a degradation rate of 12.97%, which shows that increasing the β-TCP content could accelerate the degradation rate of the scaffold in SBF solution. Figure 4B showed that after immersing in SBF solution for 6 weeks, the pH values of the scaffolds exhibited a slow downward trend, and the change in pH of the medium was not significantly different during the degradation period. The pH of the repair tissue fluid may play a regulatory role in bone healing and mineralization. It had been reported that the pH of bone repair tissue during the first week after trauma was lower than that of normal serum. The low pH and local acidic environment created at the fracture site can lead to hypoxic or ischemic conditions. The results of this experiment showed that the pH value of each group of scaffolds dropped to 6.9 and 6.8 respectively in the first 6 weeks, showing an alkaline state, which is conducive to improving the local microenvironment and conducive to cell growth.

### 3.3 Biocompatibility of the scaffolds *in vitro*

**FIGURE 5:**
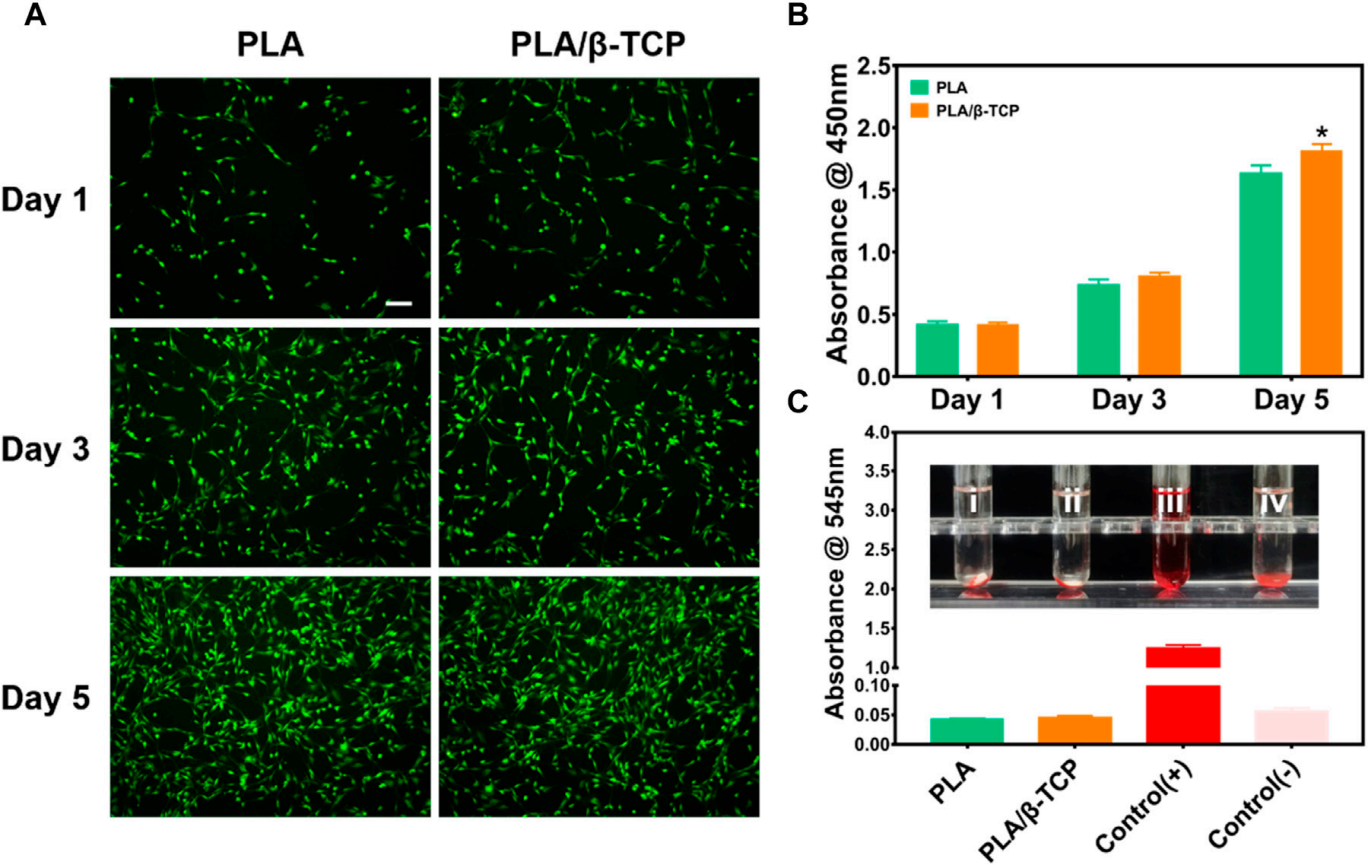
Biocompatibility of the scaffolds *in vitro*. **(A)** Fluorescence images of MC3T3-E1 cells cultured on PLA and PLA/β-TCP scaffolds for days 1, 3, and 5, respectively. Scale bar = 100 μm. **(B)** CCK-8 assay of the different scaffolds for days 1, 3, and 5. **(C)** Hemolysis test *in vitro*. Data are presented as mean ± SD (*n* = 3), *p < 0.05, **p < 0.01.

We first tested the scaffold’s biocompatibility by live/dead staining assay (Figure 5A). It can be seen that MC3T3-E1 cells in each group of scaffolds survived (green fluorescent dots) after culture for 1, 3, and 5 days, and no dead MC3T3-E1 cells (red fluorescent dots) were found. The density of viable cells gradually increased over time in the PLA and PLA/β-TCP groups, suggesting that these scaffolds had good cytocompatibility and would not inhibit the proliferation of MC3T3-E1 cells. Figure 5B was the CCK-8 assay to measure the cell proliferation of MC3T3-E1 cells co-cultured with PLA and PLA/β-TCP scaffolds, respectively. The results showed that MC3T3-E1 cells in each group showed proliferation with time. Among them, on day 1 and day 3, there was no significant difference in the proliferation of cells in each group. Interestingly, the OD values of the PLA/β-TCP group were significantly higher than that of the PLA group at day 5, which means that the PLA/β-TCP group promotes cell proliferation.

The hemocompatibility of medical materials is an essential part of evaluating the biocompatibility of medical materials, which is of great significance (Douglass et al., 2022). The hemolysis rate is an important indicator for assessing blood compatibility, and the higher the hemolysis rate, the greater the damage of the biomaterial to red blood cells (Hakimi et al., 2023). According to the standard (ISO) 10993, the hemolysis rate of medical materials <5% meets the medical standard (Liu et al., 2023). Figure 5C reflects the hemolysis of different scaffolds. As shown in the Figure 5C, it can be seen that there is no hemolysis in each group of scaffolds. The results of the hemolysis rate showed that the hemolysis rate of the tested samples was less than 5%, indicating that each group of scaffolds had good hemolytic safety. Satisfactory biomaterial biomimetic scaffolds should be non-toxic. The above results indicated that both the prepared PLA scaffold and PLA/β-TCP scaffold had good biocompatibility *in vitro*. However, the PLA/β-TCP scaffold could further promote cell proliferation.

Evaluating the biocompatibility and effectiveness of biomaterials is a crucial link for the clinical application of biomaterials. As biomaterials are increasingly widely used in the medical field, their preclinical biosafety evaluation is becoming increasingly critical, and biocompatibility evaluation has become an indispensable part of biomaterials before clinical application (da Rocha et al., 2023; Canciani et al., 2023). The degradation products of β-TCP are mainly calcium ions and phosphate ions, which are already present in our bodies.

### 3.4 Osteogenic differentiation *in vitro*

Once the bone is damaged, osteoblasts play a vital role in bone healing. After bone injury, endogenous bone marrow mesenchymal stem cells (BMSCs) will migrate to the injury site, then proliferate and further differentiate into osteoblasts. With the maturation of osteoblasts and the formation of mineralized tissue, the damaged bone is expected to heal through two pathways of intramembranous osteogenesis or endochondral osteogenesis and finally achieve the purpose of bone reconstruction (Guan et al., 2012; Takarada et al., 2016). Therefore, osteoblasts are special bone-forming cells that can synthesize bone matrix, regulate mineralization, and eventually differentiate into osteocytes, and are the most critical functional cells in the process of bone remodeling (Liu et al., 2023).

**FIGURE 6:**
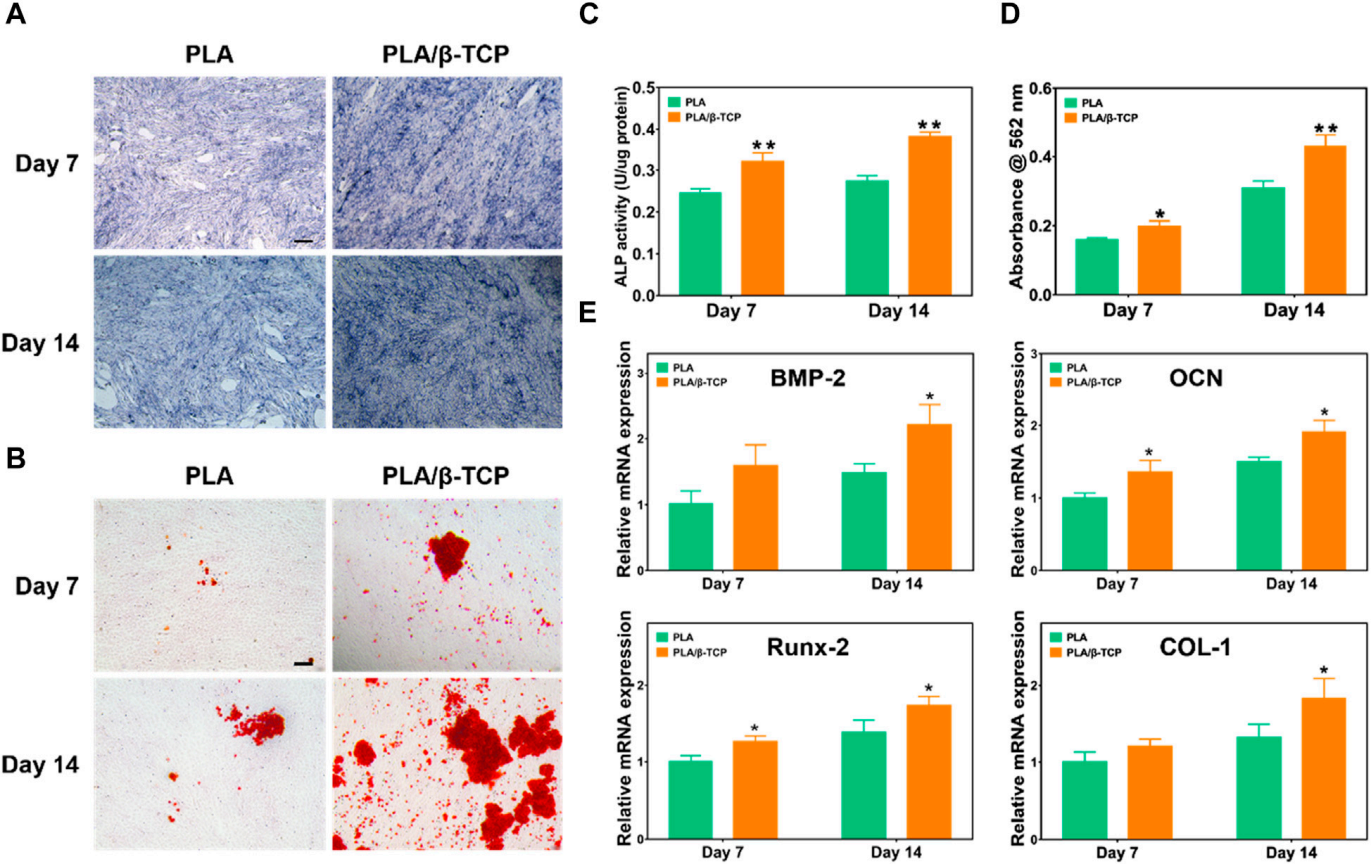
Osteogenic differentiation of the scaffolds *in vitro*. **(A)** The ALP staining of the MC3T3-E1 cells was co-cultured with the scaffolds. Scale bar = 100 μm. **(B)** Alizarin red staining of the MC3T3-E1 cells co-cultured with the scaffolds. Scale bar = 100 μm. **(C)** ALP activity of the scaffolds. **(D)** Alizarin red S quantitation of MC3T3-E1. **(E)** Relative mRNA expression of the osteogenic-related genes (BMP-2, OCN, Runx-2, and COL-1) of the MC3T3-E1 cocultured with the scaffolds. Data are presented as mean ± SD (*n* = 3). *p < 0.05, **p < 0.01.

ALP could be used as a marker to evaluate osteogenic differentiation and bone formation (Farley and Baylink, 1986; Vimalraj, 2020). Figure 6A showed the results of ALP staining of MC3T3-E1 cells. It was found that compared with the PLA group, the ALP staining color of MC3T3-E1 cells in the PLA/β-TCP was darker, and the number of blue cells was more on days 7 and 14. The ALP protein expression of the PLA/β-TCP scaffold was higher than the PLA scaffold (Figure 6C). These results indicated that more ALP was produced when MC3T3-E1 cells co-cultured with PLA/β-TCP scaffold.

Osteoblasts will produce mineralized nodules after induction and culture. Alizarin red staining can reflect the degree of calcium deposition in the extracellular matrix and then evaluate the osteogenic differentiation performance (Lee et al., 2020). We investigated cell mineralization by alizarin red S staining. Figure 6B showed representative images of Alizarin Red staining. After 7 days and 14 days of osteoinductive culture in each composition, cells cultured with PLA/β-TCP scaffolds showed larger red staining areas and more mineralized nodules compared with the PLA group. Figure 6D showed the semi-quantitative results of alizarin red mineralized nodules. The results were consistent with the staining results, the PLA/β-TCP group was higher than the PLA group (*p* < 0.05), and the differences were statistically significant, which means they were consistent with the staining results. The above results suggested that the PLA/β-TCP scaffolds help to promote the generation of MC3T3-E1 mineralized nodules, thereby promoting osteogenic differentiation.

Moreover, Figure 6E demonstrated the effect of scaffolds on the expression of important osteogenesis-related genes in MC3T3-E1 cells, including BMP-2, OCN, Runx-2, and COL-1 (Ma et al., 2018). After 7 days of intervention, the expression of OCN, Runx-2, and COL-1 mRNA in the PLA/β-TCP group was higher than that in the PLA group (*p* < 0.05). At 14 days after the intervention, the mRNA expressions of BMP-2, Col-1, and OPN in the PLA/β-TCP group were higher than those in the PLA group (*p* ≤ 0.05). The mRNA expression levels of all these osteogenic genes showed similar trends to those of cells co-cultured with PLA/β-TCP scaffolds. OCN could reflect the activity of osteoblasts and was a critical factor in the formation of mineralized bone (Liu et al., 2023). Runx2 is a member of the RUNX family of transcription factors and is involved in osteoblast differentiation and bone morphogenesis. Runx2 could regulate the transcription of different genes such as osteocalcin by binding to core sites of promoters or enhancers. Therefore, Runx2 was considered to play a crucial role in the maturation and ossification of osteoblasts (Wang et al., 2022; Ghafari et al., 2023). As a fibrous collagen, COL-1 is the most abundant and vital protein in the human body and is particularly important for developing bone tissue (Chen et al., 2021). BMP-2 can induce the directional differentiation and proliferation of undifferentiated mesenchymal stem cells into chondrocytes and osteoblasts and promote the differentiation and maturation of osteoblasts (Wang et al., 2023). This suggests that the osteogenic properties of the scaffold were enhanced after the addition of β-TCP.

**FIGURE 7:**
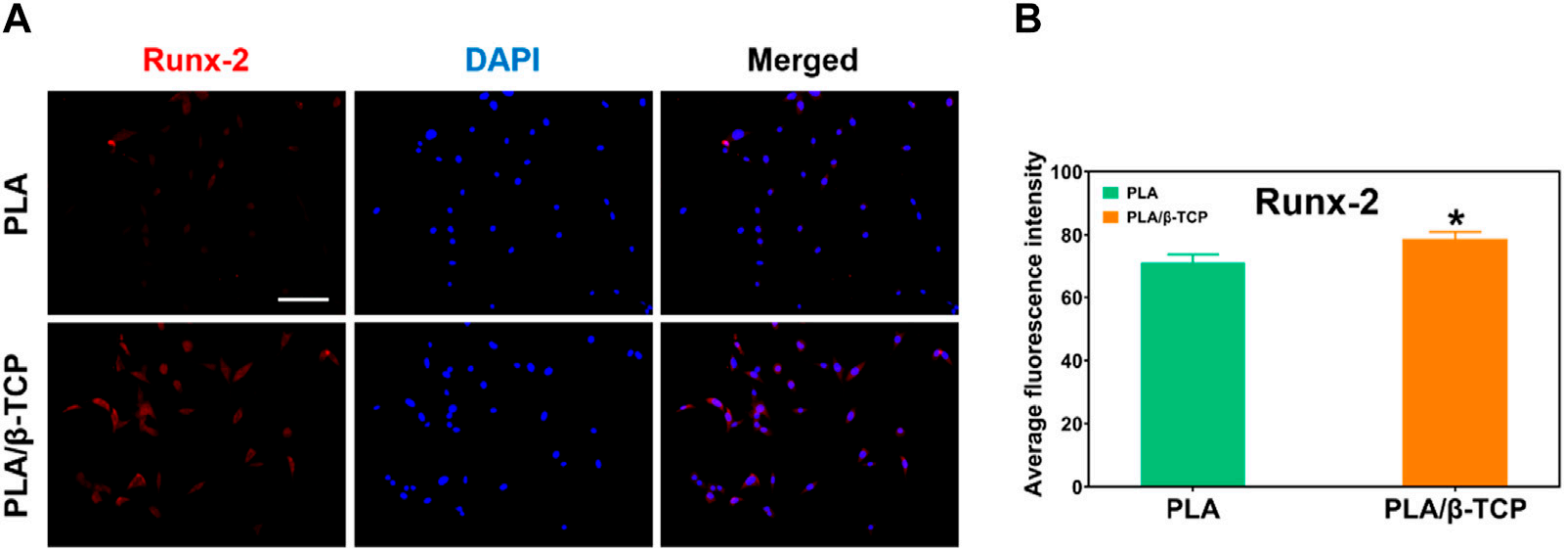
Immunofluorescence staining of osteogenic factors. **(A)** The image of immunofluorescence staining of Runx-2. Scale bar = 100 μm. **(B)** Average fluorescence intensity of Runx-2. Data are presented as mean ± SD (*n* = 3). *p < 0.05, **p < 0.01.

We further detected the osteogenic markers Runx-2 *in vitro* to evaluate the osteogenic potential of MC3T3-E1 cells cultured on different scaffolds. Figure 7 showed the immunofluorescence staining results of osteogenic markers such as Runx-2. As shown in Figure 7A, the immunofluorescence of Runx-2 in the PLA/β-TCP group was higher than in the PLA group. Figure 7B was the quantitative analysis of immunofluorescence by ImageJ. The results showed that t the fluorescence intensity of Runx-2 expression in the PLA/β-TCP group was higher than that in the PLA group (*p* ≤ 0.05), indicating that the PLA/β-TCP scaffold did have a positive effect on enhancing the expression of Runx-2.

**FIGURE 8:**
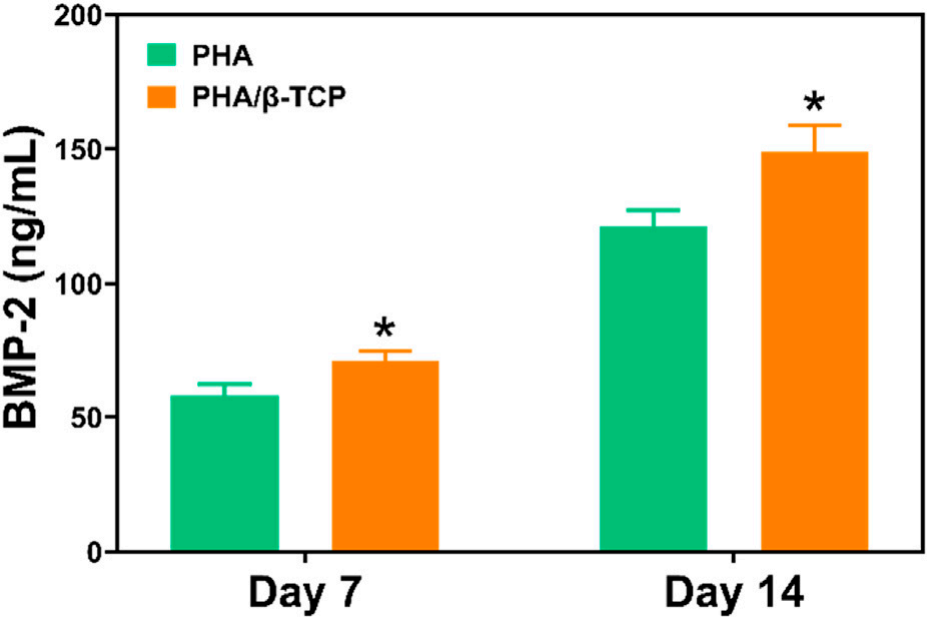
ELISA assay detects the effects of different scaffolds on BMP-2 expression. Data are presented as mean ± SD (*n* = 3). *p < 0.05, **p < 0.01.

Figure 8 showed the results of the ELISA assay of the release of BMP-2 secreted by MC3T3-E1 cells after 7 and 14 days of intervention with different scaffolds. As shown in Figure 8, BMP-2 levels continued to increase in different scaffolds on days 7 and 14. In contrast, the expression level of BMP-2 in MC3T3-E1 cells in the PLA/β-TCP group was significantly higher than that in the PLA group (*p* ≤ 0.05). Therefore, the concentration of this bone-related specific protein marker was relatively higher in the PLA/β-TCP group, this result was consistent with the results of RT-PCR.

Seed cells, bioactive molecules, and scaffolds were the cornerstones of BTE. A wide variety of biomaterials were considered promising candidates for bone regeneration. Bioceramics including hydroxyapatite (HA), β-TCP, and bioglasses (BGs) were thought to be bioactive in forming new bone (Koons et al., 2020). β-TCP had a solubility more similar to that of bone minerals than to HA, making it more readily absorbed and replaced by new bone. However, HA was more crystalline and therefore more difficult to degrade *in vivo* and more likely to cause bone deformities and fractures around hydroxyapatite bone grafts (Ogose et al., 2005). BG has strong osteogenic potential. However, BG has limitations such as inflammation and infection, which limits its further application (Sivakumar et al., 2023). PLA was less osteoconductive or osteoinductive, but β-TCP can effectively promote osteogenesis, mainly because tricalcium phosphate can trigger the differentiation of stem cells/osteogenic cells into osteoblasts, the release of calcium ions and phosphate ions It also had strong cell chemotaxis, and can recruit various types of cells to grow to the implantation site, thereby promoting bone tissue regeneration (Lai et al., 2019). Besides, the photosensitive material was loaded into the LCD printer in liquid form, making it easier and faster to prepare complex geometric shapes than traditional FDM molten plastic filaments, and the surface of the prepared scaffold was smoother. In summary, considering the proliferation and osteogenic differentiation of PLA/β-TCP scaffolds using LCD technology on MC3T3-E1 cells, we believe 3D printing of PLA/β-TCP scaffolds by LCD technology will play a potentially important role in bone repair. Taken together, the above data suggest that the PLA/β-TCP scaffold by LCD technology could enhance bone mineralization and osteogenic differentiation.

## 4 Conclusion

In summary, we successfully designed and fabricated an innovative 3D printed PLA/β-TCP scaffold using LCD photocuring and further evaluated the physical properties, biocompatibility, and *in vitro* osteogenic performance of the PLA/β-TCP scaffold. SEM results show that the prepared PLA/β-TCP scaffolds containing different proportions of β-TCP all have three-dimensional interconnected network structures. The compressive strength test results show that when the β-TCP dosage is 10%, the compressive strength of PLA/TCP reaches the maximum value of 52.1 MPa. In addition, the 3D printed PLA/β-TCP scaffold not only has a more suitable physical structure, but also provides more suitable mechanical properties, and also has great potential for bone regeneration. Live/dead staining assay and hemocompatibility assay demonstrated that the prepared PLA/β-TCP scaffold had good biocompatibility. CCK-8 assay showed that the PLA/β-TCP group promotes cell proliferation. More importantly, ALP staining assay, ALP activity, Alizarin red stained, RT-qPCR and Immunofluorescence staining show that the prepared PLA/β-TCP scaffold showed great potential to promote osteogenic differentiation of MC3T3-E1 cells *in vitro*. Therefore, 3D printed LCD photocuring PLA/β-TCP scaffolds can undoubtedly improve surface bioactivity and lead to better osteogenesis, which may provide a unique strategy for developing bioactive implants in orthopedic applications.

## Data Availability

The raw data supporting the conclusion of this article will be made available by the authors, without undue reservation.
