# Supplementary material for: Fabrication and properties of PLA/β-TCP scaffolds using liquid crystal display (LCD) photocuring 3D printing for bone tissue engineering
Source: Front Bioeng Biotechnol. 2024 Feb 19;12:1273541. doi: 10.3389/fbioe.2024.1273541 (PMC10910430; doi:10.3389/fbioe.2024.1273541)
Supplement: Supplementary file 1 [file DataSheet1.docx]

Supporting Information

**Fabrication and properties of** **PLA/β-TCP scaffolds using** **liquid crystal display (LCD)** **photocuring** **3D printing for bone tissue engineering**

Boqun Wang*^1,2#^*, Xiangling Ye*^3,4#^*, Guocai Chen^5^*^#^*, Yongqiang Zhang*^4^*, Zhikui Zeng*^6^*, Cansen Liu*^1^*, Zhichao Tan*^3*^*, Xiaohua Jie*^1*^*,

*1 School of Materials and Energy, Guangdong University of Technology, Guangzhou, Guangdong, 510006, PR China*

*2 School of Intelligent Manufacturing, Dongguan Polytechnic, Dongguan, Guangdong, 523808, PR China*

*3 Dongguan Hospital, Guangzhou University of Chinese Medicine, Dongguan, Guangdong, 523005, PR China*

*4* *The Second Clinical College of Guangzhou University of Chinese Medicine, Guangzhou, Guangdong, 510405, PR China*

*5 Foshan Hospital of Traditional Chinese Medicine, Guangzhou University of Chinese Medicine, Foshan Guangdong, 528000, PR China.*

*6 Affiliated Hospital of Jiangxi University of Chinese Medicine, Nanchang, Jiangxi, 330006, PR China*

****Correspondence and requests for materials should be addressed to:***

*Xiaohua Jie, Ph.D. (email: jiexh@gdut.edu.cn) School of Materials and Energy, Guangdong University of Technology, Guangzhou Guangdong, PR China, 510006*

*Zhichao Tan, MD. (email:* [*tanzhichao73@126.com*](mailto:tanzhichao73@126.com)*) Dongguan Hospital, Guangzhou University of Chinese Medicine, Dongguan, Guangdong, 523005, PR China*

***^#^ These authors contributed equally.***


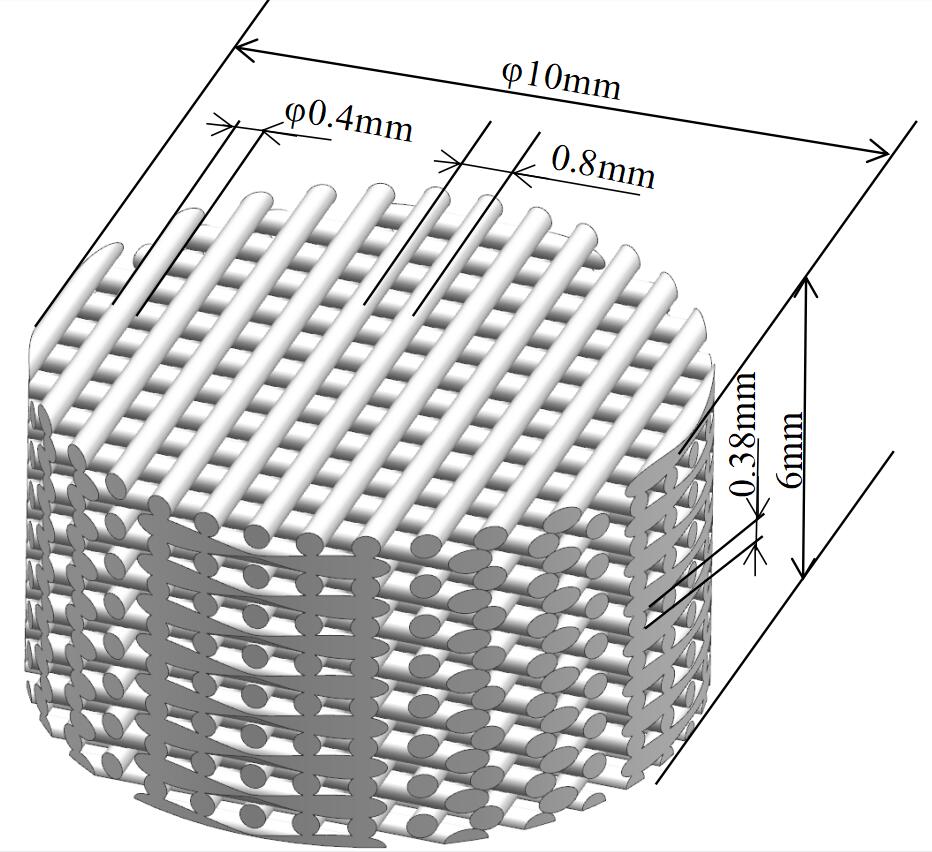


Figure 1. Schematic diagram of the scaffold structure

Table S1 LCD 3D printing parameters

| parameter name | Values |
| --- | --- |
| Layer Height (mm) | 0.05 |
| Bottom Lift Distance (mm) | 5 |
| Lifting Distance (mm) | 5 |
| Bottom Layer Count (Layer) | 2 |
| Exposure Time (s) | 1 |
| Bottom Exposure Time (s) | 12 |
| Bottom Lift Speed (mm/min) | 50 |
| Lifting Speed (mm/min) | 50 |
| Retract Speed (mm/min) | 150 |
| Light-off Delay (s) | 0 |
| Bottom Light-off Delay (s) | 0 |

Table S2 Primer sequences for RT-qPCR

| Gene | Primer sequences | |
| --- | --- | --- |
|  | Forward (5′-3′) | Reverse (5′-3′) |
| GAPDH  RUNX-2  Col-1α  OCN  BMP-2 | CATGGCCTTCCGTGTTCCTA  TCCAACCCACGAATGCACTA  GCAGGGTTCCAACGATGTTG  CAATAAGGTAGTGAACAGAC  GAACACAAGTCAGTGGGAGAG | CCTGCTTCACCACCTTCTTGAT  GAAGGGTCCACTCTGGCTTTG  AGGAACGGCAGGCGAGAT  CTTCAAGCCATACTGGTCT  CACCTGGGTTCTCCTCTAAATG |


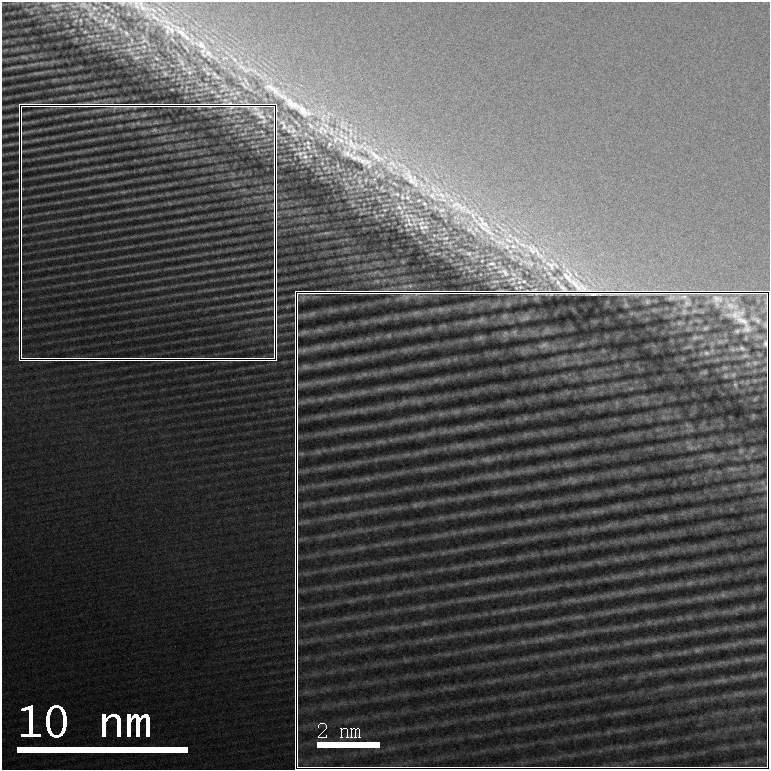

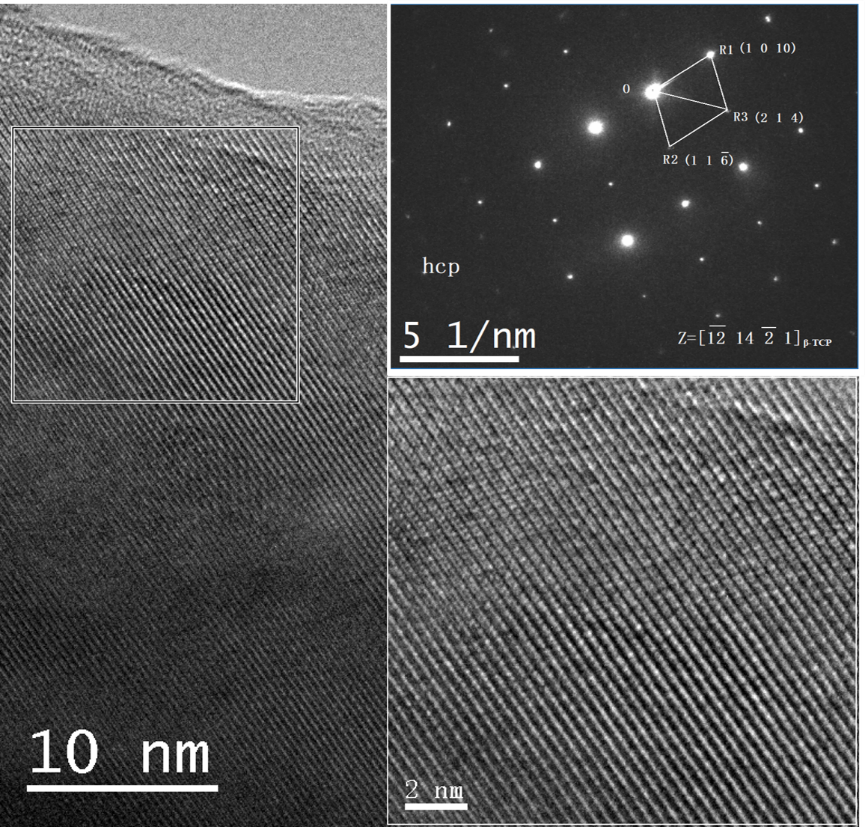


Figure S2. Selected Area Electron Diffraction (SAED) pattern of Particle

Figure S3. Porosity of Scaffolds


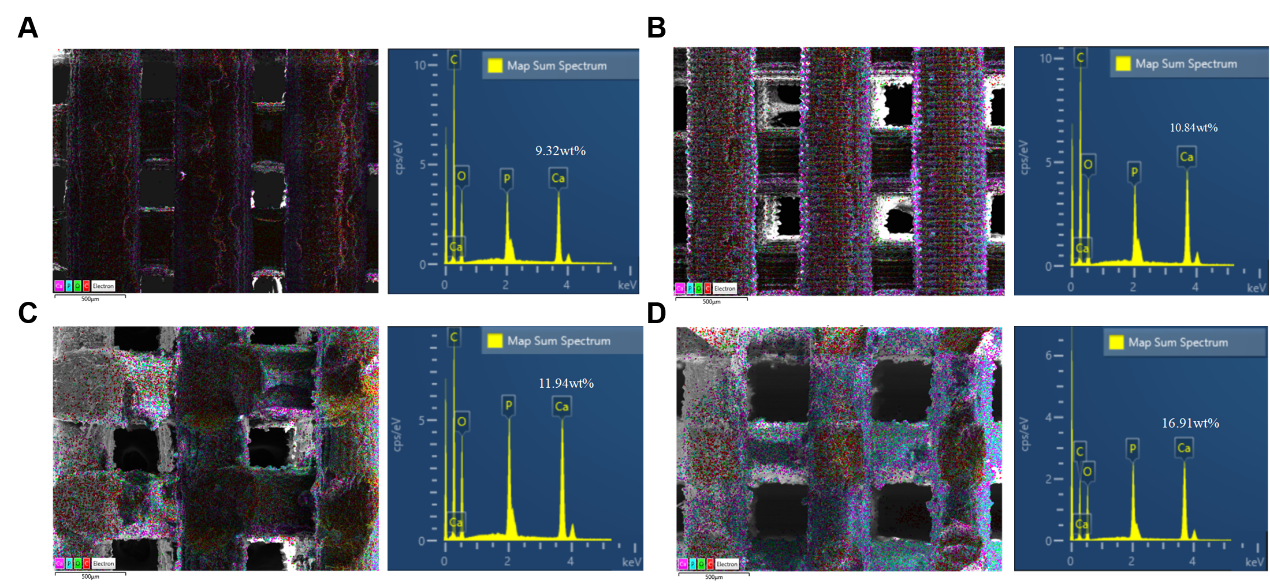


Figure S4. The weight ratio of Ca in different scaffolds surface scanning. (a) PLA/10%β-TCP, (b)PLA/20%β-TCP, (c) PLA/30%β-TCP, (d)PLA/35%β-TCP

Figure S5. X-ray diffraction analysis was performed on β-TCP powder and PLA/10%β-TCP scaffold
